# Effect of focal adhesion kinase inhibition on osteoblastic cells grown on titanium with different topographies

**DOI:** 10.1590/1678-7757-2019-0156

**Published:** 2020-01-31

**Authors:** Helena Bacha LOPES, Alann Thaffarell Portilho SOUZA, Gileade Pereira FREITAS, Carlos Nelson ELIAS, Adalberto Luiz ROSA, Marcio Mateus BELOTI

**Affiliations:** 1 Universidade de São Paulo Faculdade de Odontologia de Ribeirão Preto Bone Research Laboratory Ribeirão PretoSão Paulo Brasil Universidade de São Paulo, Faculdade de Odontologia de Ribeirão Preto, Bone Research Laboratory, Ribeirão Preto, São Paulo, Brasil.; 2 Instituto Militar de Engenharia Laboratório de Biomateriais Rio de JaneiroRio de Janeiro Brasil Instituto Militar de Engenharia, Laboratório de Biomateriais, Rio de Janeiro, Rio de Janeiro, Brasil.

**Keywords:** Bone, Focal adhesion kinase, Osteoblast, Titanium

## Abstract

**Objective:**

The present study aimed to investigate the participation of focal adhesion kinases (FAK) in interactions between osteoblastic cells and titanium (Ti) surfaces with three different topographies, namely, untreated (US), microstructured (MS), and nanostructured (NS).

**Methodology:**

Osteoblasts harvested from the calvarial bones of 3-day-old rats were cultured on US, MS and NS discs in the presence of PF-573228 (FAK inhibitor) to evaluate osteoblastic differentiation. After 24 h, we evaluated osteoblast morphology and vinculin expression, and on day 10, the following parameters: gene expression of osteoblastic markers and integrin signaling components, FAK protein expression and alkaline phosphatase (ALP) activity. A smooth surface, porosities at the microscale level, and nanocavities were observed in US, MS, and NS, respectively.

**Results:**

*FAK* inhibition decreased the number of filopodia in cells grown on US and MS compared with that in NS. FAK inhibition decreased the gene expression of Alp, bone sialoprotein, osteocalcin, and ALP activity in cells grown on all evaluated surfaces. *FAK* inhibition did not affect the gene expression of Fak, integrin alpha 1 ( *Itga1* ) and integrin beta 1 ( *Itgb1* ) in cells grown on MS, increased the gene expression of Fak in cells grown on NS, and increased the gene expression of *Itga1* and *Itgb1* in cells grown on US and NS. Moreover, FAK protein expression decreased in cells cultured on US but increased in cells cultured on MS and NS after FAK inhibition; no difference in the expression of vinculin was observed among cells grown on all surfaces.

**Conclusions:**

Our data demonstrate the relevance of FAK in the interactions between osteoblastic cells and Ti surfaces regardless of surface topography. Nanotopography positively regulated FAK expression and integrin signaling pathway components during osteoblast differentiation. In this context, the development of Ti surfaces with the ability to upregulate FAK activity could positively impact the process of implant osseointegration.

## Introduction

Excellent mechanical and biological properties render titanium (Ti) to be the most frequently used biomaterial for manufacturing dental implants.^1^ The bone−Ti contact is influenced by several parameters, including topography and surface chemistry of the dental implants. Osseointegration of implants is strongly associated with the responses of osteoblasts to the surface of the biomaterial, and signaling pathways involved in osteoblastic differentiation, such as integrin signaling, are known to play an important role in this process.

Integrins are heterodimeric transmembrane proteins composed of α and β subunits forming a family of membrane receptors whose primary function is adhesion of cells to extracellular matrix proteins, such as collagen and fibronectin; some of these receptors involved in osteoblastic differentiation.^1 - 3^ The integrin signaling pathway acts in both directions of the cell membrane, i.e., binding of integrins to components of the extracellular matrix triggers an intracellular signaling cascade and the activation of integrins is modulated by intracellular signals and, consequently, their affinity to the ligand present in the extracellular matrix.^4^

Focal adhesion kinases (FAK) or Src family kinases are the main integrin-activated protein tyrosine kinases that play a key role in this signaling pathway.^1 , 4 - 6^ The associations of extracellular matrix ligands, integrins, and cytoskeletal components form a focal adhesion complex, where FAK is recruited and interacts directly or indirectly with these complexes causing their activation by autophosphorylation and consequent binding to Src kinase. Src kinases phosphorylate several components of focal adhesion sites participating in FAK signaling to generate the signal transduction mechanism.^7 , 8^

The development of biomaterials modulating the interaction between integrins and the extracellular matrix represents an important strategy for therapies related to bone tissue in dentistry and medicine. Several studies have demonstrated the participation of the integrin signaling pathway, through FAK, in response of osteoblastic cells to different surface topographies.^9 - 16^ Thus, the role of specific FAK inhibitors in the osteogenic potential of cells on Ti surfaces is an interesting research topic. The present study aimed to investigate the participation of FAK in interactions between osteoblastic cells and Ti surfaces with different topographies by using a specific FAK inhibitor.

## Methodology

### Cell culture

Osteoblastic cells were obtained according to a method previously described.^17^ All procedures involving animals were approved by the animal care guidelines of the School of Dentistry of Ribeirão Preto (CEUA/FORP Protocol #2015.1.581.58.1). Briefly, 3-day-old Wistar rats were decapitated with a scalpel blade, and their calvaria were removed with the use of scissors. Osteoblastic cells were isolated through sequential enzymatic digestion with 0.25% trypsin solution (Gibco-Invitrogen, Grand Island, NY, USA) and collagenase type II 0.20% (Gibco-Invitrogen). Only cells isolated in the 2^nd^ and 3^rd^ digestion were used.

### Selection of FAK inhibitor concentration

A potent and specific FAK inhibitor, PF-573228 (3,4-dihydro-6-[[4-[[[3-(methylsulfonyl) phenyl] methyl] amino]-5-(trifluoromethyl)-2-pyrimidinyl] amino]-2(1H)-quinolinone) (Sigma-Aldrich, Darmstadt, Germany), which interacts with ATP binding sites and blocks their catalytic activity, was used in this study.^18 - 20^ PF-573228 was reconstituted in dimethylsulfoxide (DMSO) (Sigma-Aldrich) to produce a 20 mg/mL stock solution, which was then diluted to final concentrations of 0.1, 1 and 10 µM according to a previous study.^20^ Next, a non-cytotoxic concentration of PF-573228 was determined. Here, osteoblastic cells were cultured on Thermanox^®^ coverslips (Nunc, Rochester, NY, USA) at a density of 1×10^4^ cells/well in 24-well polystyrene plates (Corning Incorporated, Corning, NY, USA) containing osteogenic medium (OM) prepared with alpha-minimum essential medium (α-MEM, Gibco-Invitrogen), 10% fetal calf serum (Gibco-Invitrogen), 50 µg/mL gentamicin (Gibco-Invitrogen), 0.3 mg/mL fungisone (Gibco-Invitrogen), 5 μg/mL ascorbic acid (Gibco-Invitrogen), and 7 mM β-glycerophosphate (Sigma-Aldrich) for up to 3 days and exposed to three different concentrations of PF-573228 or to vehicle (DMSO, control). On day 3, cells were counted by staining of cell nuclei with 300 nM 4′,6-diamidino-2-phenylindole, dihydrochloride (DAPI; Molecular Probes, Thermo Fischer Scientific, Waltham, MA, USA) as described elsewhere.^21^ An AxioCam MRM digital camera (Carl Zeiss Inc., Oberkochen, Germany) coupled with an AxioImager M2 Zeiss light microscope (Carl Zeiss Inc.) was used to analyze the samples. The cell nuclei were counted from three different fields on three different coverslips for each treatment (n=9).

### Preparation of Ti surfaces

Discs of pure grade 2 Ti (Realum, São Paulo, SP, Brazil; diameter, 12 mm; thickness, 1.5 mm) were polished with up to 600 grit silicon carbide and treated with either HNO_3_-H_2_SO_4_-HCl to produce the microstructured surface (MS) or H_2_SO_4_-H_2_O_2_ to produce the nanostructured surface (NS).^22 , 23^ Ti with an untreated surface (US) was used as the control. All samples were rinsed several times with deionized H_2_O, air-dried, and then autoclaved. The surfaces were evaluated by field emission scanning electron microscopy (SEM) operated at 5 kV (Inspect S50, FEI, Hillsboro, OR, USA).

### Effect of FAK inhibition in osteoblastic cells grown on Ti surfaces

The cells were plated on the Ti surfaces at a density of 2×10^4^ cells/disc in 24-well polystyrene plates (Corning Incorporated) for up to 10 days, using OM containing PF-573228 0.1 µM, which was selected in a previous experiment. The parameters described below were then evaluated.

### Scanning electron microscopy

Exactly 24 h after culture on the Ti surface, SEM was carried out to observe cells grown with or without PF-573228. The samples were fixed in 4% glutaraldehyde (Electron Microscopy Sciences, Washington, PA, USA), rinsed with 0.2 M sodium cacodylate buffer, pH 7.4 (Sigma-Aldrich), post-fixed with 1% osmium tetroxide (Sigma-Aldrich), and then rinsed in sodium cacodylate buffer. Thereafter, the cells were immersed in a solution of 1% tannic acid (Sigma-Aldrich) and 0.1 M sodium cacodylate solution for 60 min and rinsed with 0.2 M sodium cacodylate. The samples were dehydrated by crescent alcohol concentrations followed by hexamethyldisiloxane prior to sputter-coating (20 nm gold/palladium) and evaluation by using a Zeiss Sigma FE-SEM microscope (Carl Zeiss Inc.).

### Gene expression of osteoblastic markers and integrin signaling pathway components

**Figure 1:**
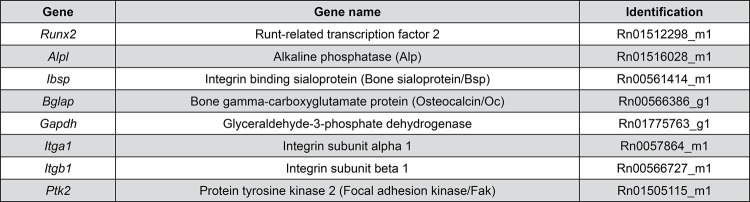
TaqMan (Life Technologies-Invitrogen) probes for real-time PCR

On day 10 after culture, quantitative real-time PCR was performed, using TaqMan (Life Technologies-Invitrogen, Carlsbad, CA, USA) probes ( Figure 1 ) to evaluate the gene expression of the osteoblastic markers alkaline phosphatase ( *Alp* ), bone sialoprotein ( *Bsp* ), and osteocalcin ( *Oc* ), as well as that of the integrin signaling pathway components integrin β1 ( *Itgb1* ), integrin α1 ( *Itga1* ), and *Fak* . The total RNA of cells cultured on Ti surfaces with or without PF-573228 was extracted to synthesize complementary DNA (cDNA) as previously described.^9^ The expression of the housekeeping gene β-actin was selected to normalize the expression of genes of interest and it was relative to the cells cultured on US, MS, or NS surfaces in the absence of PF-573228 (control) using the comparative threshold 2^-^ method.^24^

### ALP activity

On day 10, cells cultured on Ti surfaces with or without PF-573228 were lysed, and a commercial kit (Labtest Diagnostica, Lagoa Santa, MG, Brazil) was used to detect their ALP activity as previously described.^25^ The absorbance (n=4) was evaluated at 590 nm by using the plate reader μQuant (Bio-Tek Instruments Inc., Winooski, VT, USA), and ALP activity was calculated and expressed as μmol of thymolphthalein/h/mg protein as previously described.

### FAK protein detection

On day 10, cells cultured on Ti surfaces with or without PF-573228 were lysed, and the protein of each cell was extracted and transferred to PVDF membrane as previously described^9^ to evaluate FAK protein expression by Western Blotting assay. Blocking of non-specific sites was performed with 5% Non-Fat Dry Milk Blotting Grade Blocker (Bio-Rad Laboratories, Hercules, CA, USA) for 2 h. Cells were incubated overnight at 4°C with a rabbit polyclonal antibody to FAK (1:1000, Cell Signaling Technology, Denver, MA, USA) and a mouse anti-glyceraldehyde-3-phosphate dehydrogenase (GAPDH) monoclonal antibody (1:2000, Santa Cruz Biotechnology), which was used as a control. Secondary antibodies conjugated to HRP (1:2000, Santa Cruz Biotechnology) were used for immunodetection with Western Lightning Chemiluminescence Reagent (PerkinElmer Life Sciences, Waltham, MA, USA), and images were captured by a G:Box gel imaging system (Syngene, Cambridge, UK).

### Statistical analysis

The data of cell counting were analyzed by a one-way analysis of variance, followed by the Student−Newman−Keuls *post hoc* test. Student’s *t* -test was used to analyze the data of gene expression and ALP activity. The significance level used was set to 5% (p≤0.05).

## Results

### Selection of FAK inhibitor concentration

**Figure 2:**
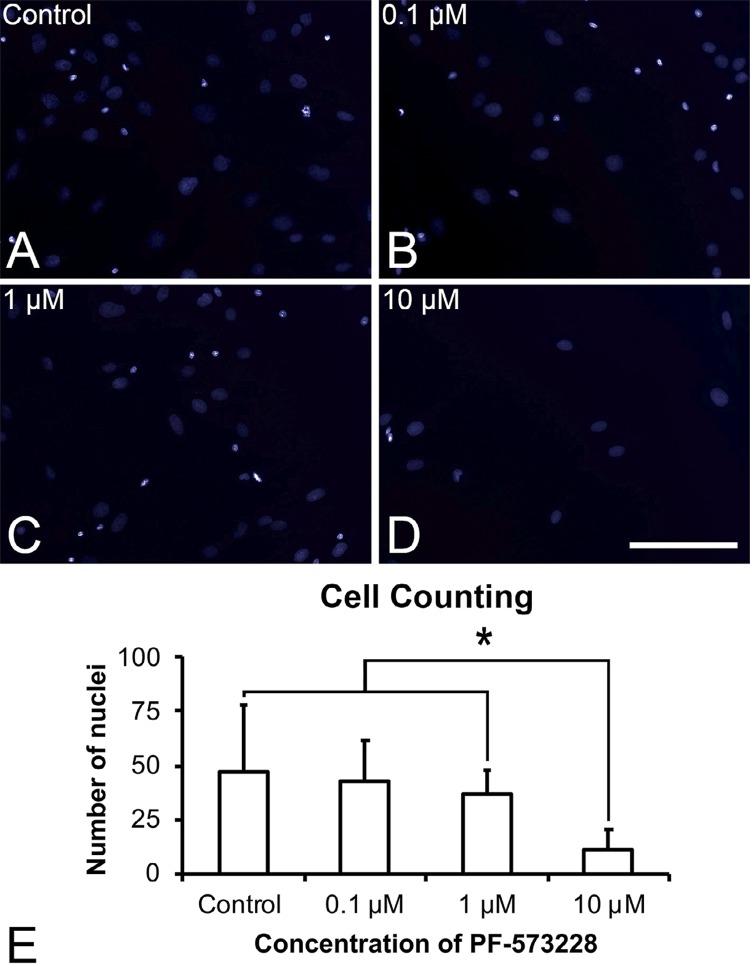
Cell counting of DAPI-positive cells in the control (A), 0.1 µM (B), 1µM (C), and 10 µM (D) PF-573228. The data (E) are presented as mean ± standard deviation (n=9), and asterisks (*) indicate statistically significant differences (p≤0.05). Scale bar: 200 µm

Fluorescence labeling of cell nuclei stained with DAPI in the control treatment ( Figure 2A ) was similar to those of cultures grown in the presence of 0.1 μM ( Figure 2B ) and 1 μM ( Figure 2C ) PF-573228 but lower in cultures grown in 10 μM PF-573228 ( Figure 2D ). Quantification of stained nuclei ( Figure 2E ) showed a statistically significantly lower number of nuclei in cells treated with 10 μM PF-573228 compared with those in cells from the control, 1 μM PF-573228, and 0.1 μM PF-573228 treatments (p=0.001, p=0.003, and p=0.046, respectively), among which no statistically significant difference was found (p>0.05). Based on this finding and taking into account that 0.1 μM is the lowest concentration of the FAK inhibitor that did not induce cytotoxic effects, further experiments were carried out using 0.1 μM PF-573228.

### Ti surface characteristics

**Figure 3:**
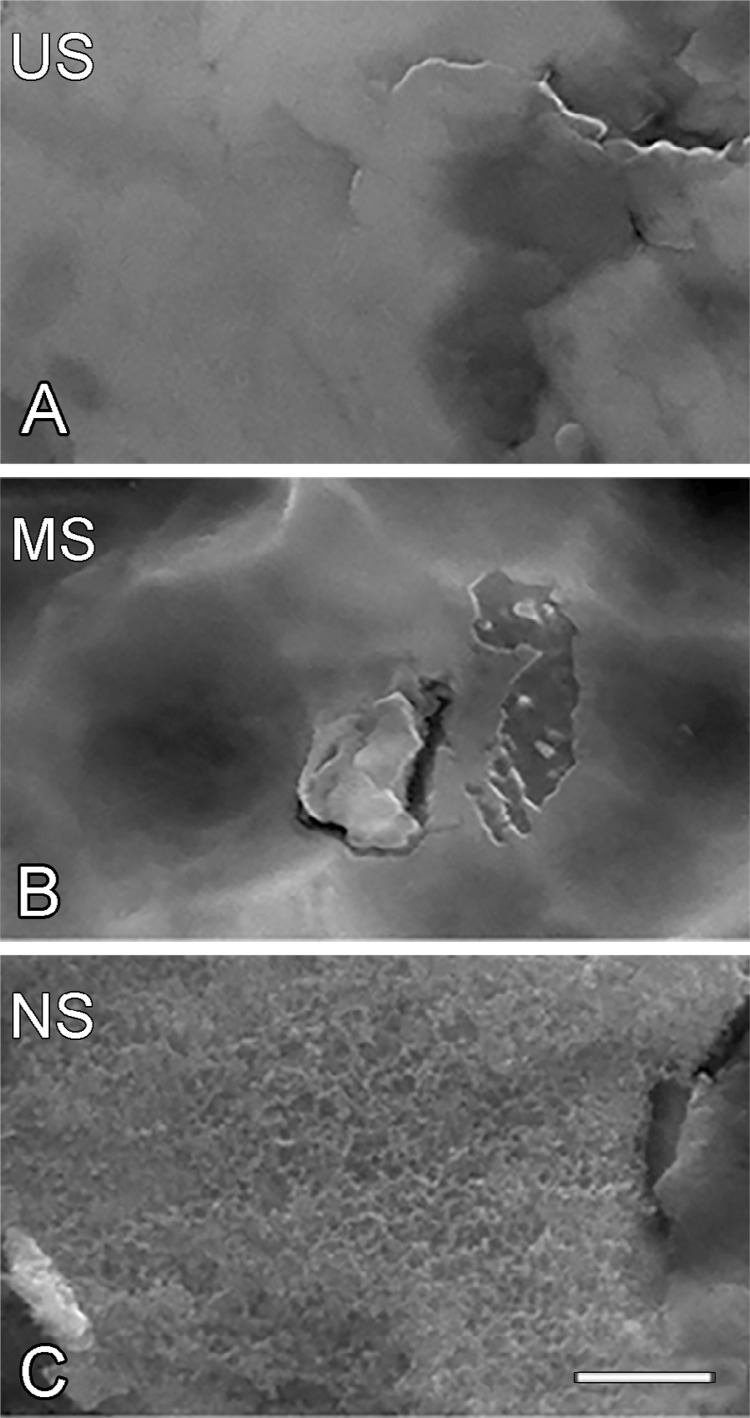
High-resolution scanning electron micrographs of US (A), MS (B), and NS (C). Scale bar: 500 nm

The methods used in this experiment effectively produced surfaces with different topographies ( Figure 3 ). US exhibited a smooth surface ( Figure 3A ), MS presented porosities at the microscale level ( Figure 3B ), and NS presented nanocavities ( Figure 3C ).

### Effect of FAK inhibition on morphology of osteoblastic cells grown on Ti surfaces

**Figure 4:**
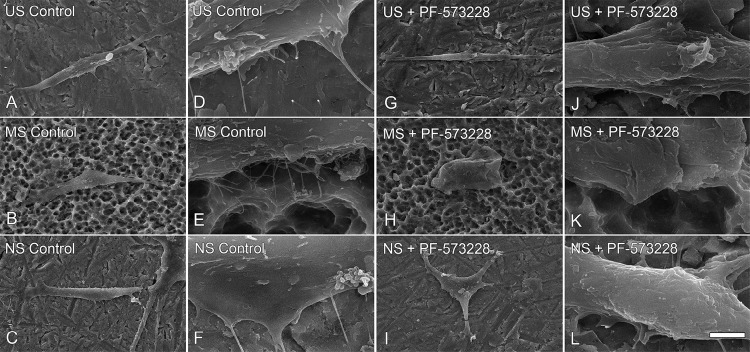
High-resolution scanning electron micrographs of cells cultured on US (A, D, G, J), MS (B, E, H, K), and NS (C, F, I, L) in presence or absence of 0.1 µM PF-573228 at 24 h. Scale bars: A, B, C, G, H, and I=10 µm; D, E, F, J, K, and L=1 µm

SEM imaging showed that the cells remained viable and well spread in all Ti surfaces ( Figure 4A-C ), with higher number of filopodia on them ( Figure 4D-F ). FAK inhibition decreased the number of filopodia in cells grown on US and MS ( Figures 4G and 4H , respectively, and Figures 4J and 4K , respectively) but demonstrated no change in cells grown on NS ( Figures 4 and 4L ).

### Effect of FAK inhibition on the gene expression of osteogenic markers of osteoblastic cells grown on Ti surfaces

**Figure 5:**
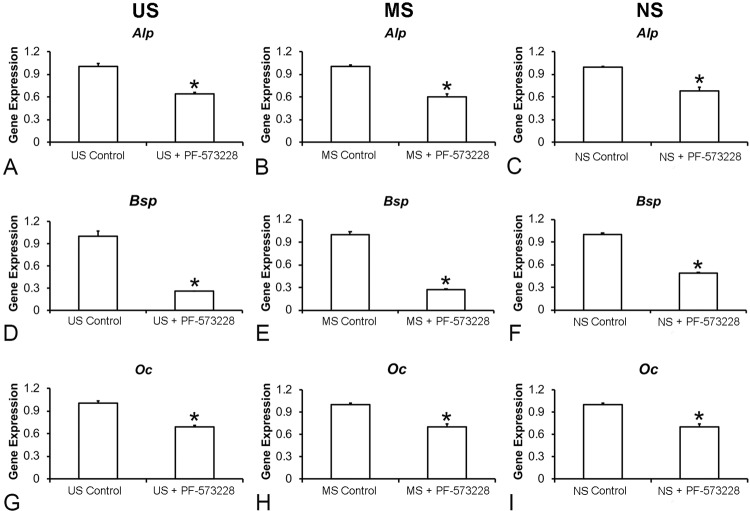
Gene expression of the osteoblastic markers alkaline phosphatase (Alp, A−C), bone sialoprotein (Bsp, D−F), and osteocalcin (Oc, G−I) in cells cultured on US, MS, and NS in presence or absence of 0.1 µM PF-573228 on day 10. The data are presented as mean ± standard deviation (n=3), and asterisks (*) indicate statistically significant differences (p≤0.05)

FAK inhibition reduced the gene expression of all evaluated osteoblastic markers in cells cultured on US, MS, and NS ( Figure 5 ). Specifically, FAK inhibition decreased the gene expression of *Alp* ( Figures 5A−5C , p=0.001 for all surfaces), *Bsp* ( Figures 5D−5F , p=0.001 for all surfaces), and *Oc* ( Figures 5G−5I , p=0.001 for US and MS; p=0.002 for NS) in cells grown on all evaluated surfaces.

### Effect of FAK inhibition on ALP activity of osteoblastic cells grown on Ti surfaces

**Figure 6:**
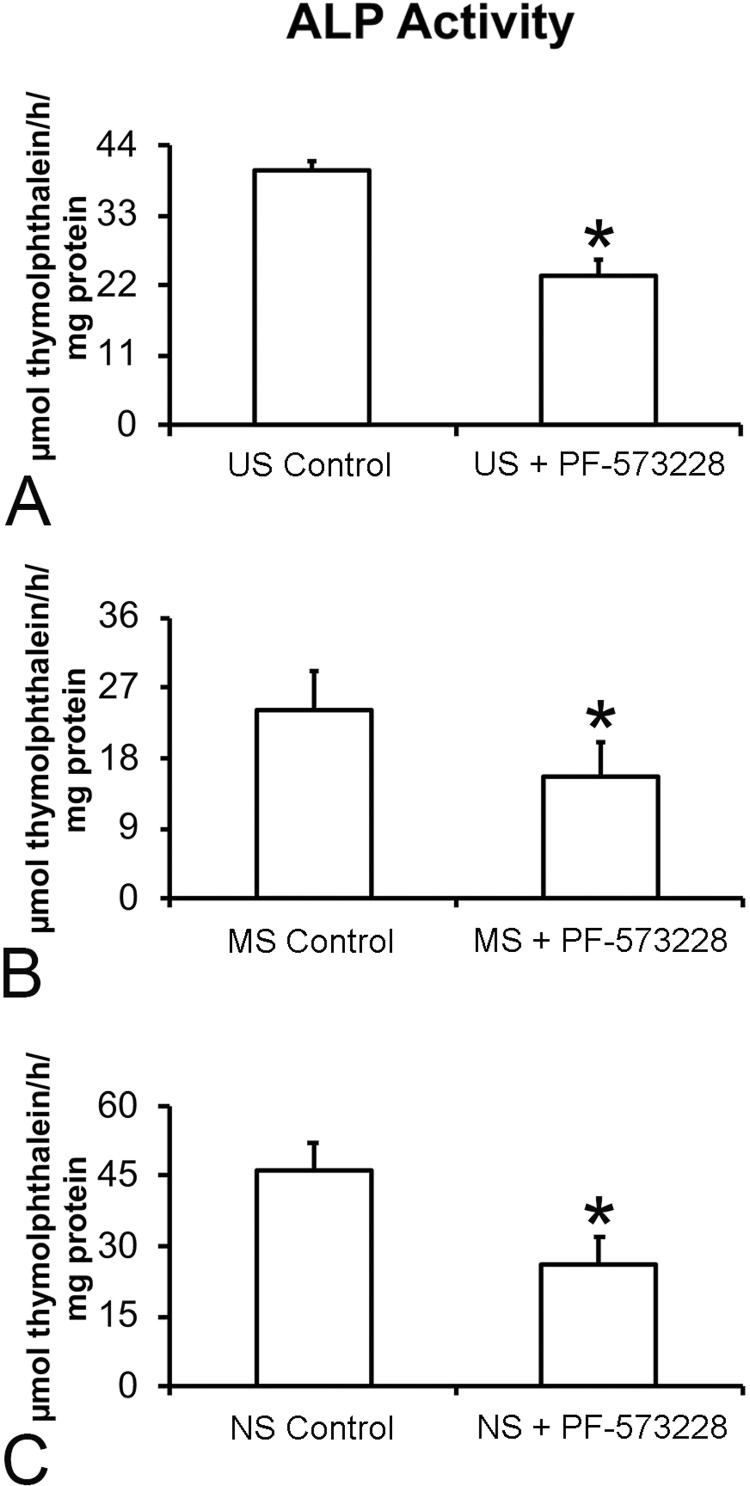
Alkaline phosphatase (ALP) activity of cells cultured on US (A), MS (B), and NS (C) in the presence or absence of 0.1 µM PF-573228 on day 10. The data are presented as mean ± standard deviation (n=3), and asterisks (*) indicate statistically significant differences (p≤0.05)

Similar to the gene expression findings, FAK inhibition decreased the ALP activity in cells grown on US ( Figure 6A , p=0.001), MS ( Figure 6B , p=0.013), and NS ( Figure 6C , p=0.001).

### Effect of FAK inhibition on the gene expression of integrin signaling pathway components in osteoblastic cells grown on Ti surfaces

**Figure 7:**
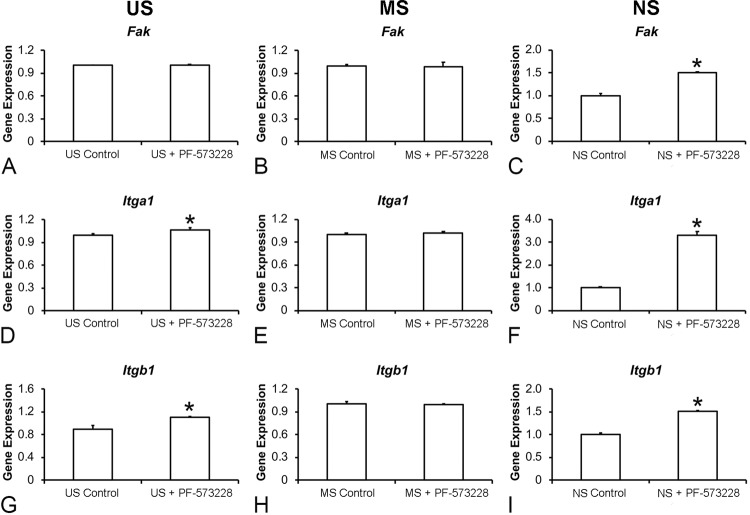
Gene expression of the integrin signaling pathway components focal adhesion kinase (Fak, A), integrin a1 (Itga1, B), and integrin b1 (Itgb1, C) in cells cultured on US, MS, and NS in presence or absence of 0.1 µM PF-573228 on day 10. The data are presented as mean ± standard deviation (n=3), and asterisks (*) indicate statistically significant differences (p≤0.05)

FAK inhibition did not affect the gene expression of *Fak* ( Figure 7A , p=0.249) but increased the gene expression of *Itga1* ( Figure 7D , p=0.015) and *Itgb1* ( Figure 7G , p=0.005) in cells cultured on US. FAK inhibition did not affect the gene expression of *Fak* ( Figure 7B , p=0.708), *Itga1* ( Figure 7E , p=0.176), and *Itgb1* ( Figure 7H , p=0.835) in cells cultured on MS. Finally, FAK inhibition increased the gene expression of *Fak* , *Itga1* , and *Itgb1* in cells cultured on NS ( Figures 7C , 7F, and 7I , respectively, p=0.001 for all genes).

### Effect of FAK inhibition on FAK protein expression in osteoblastic cells grown on Ti surfaces

**Figure 8:**
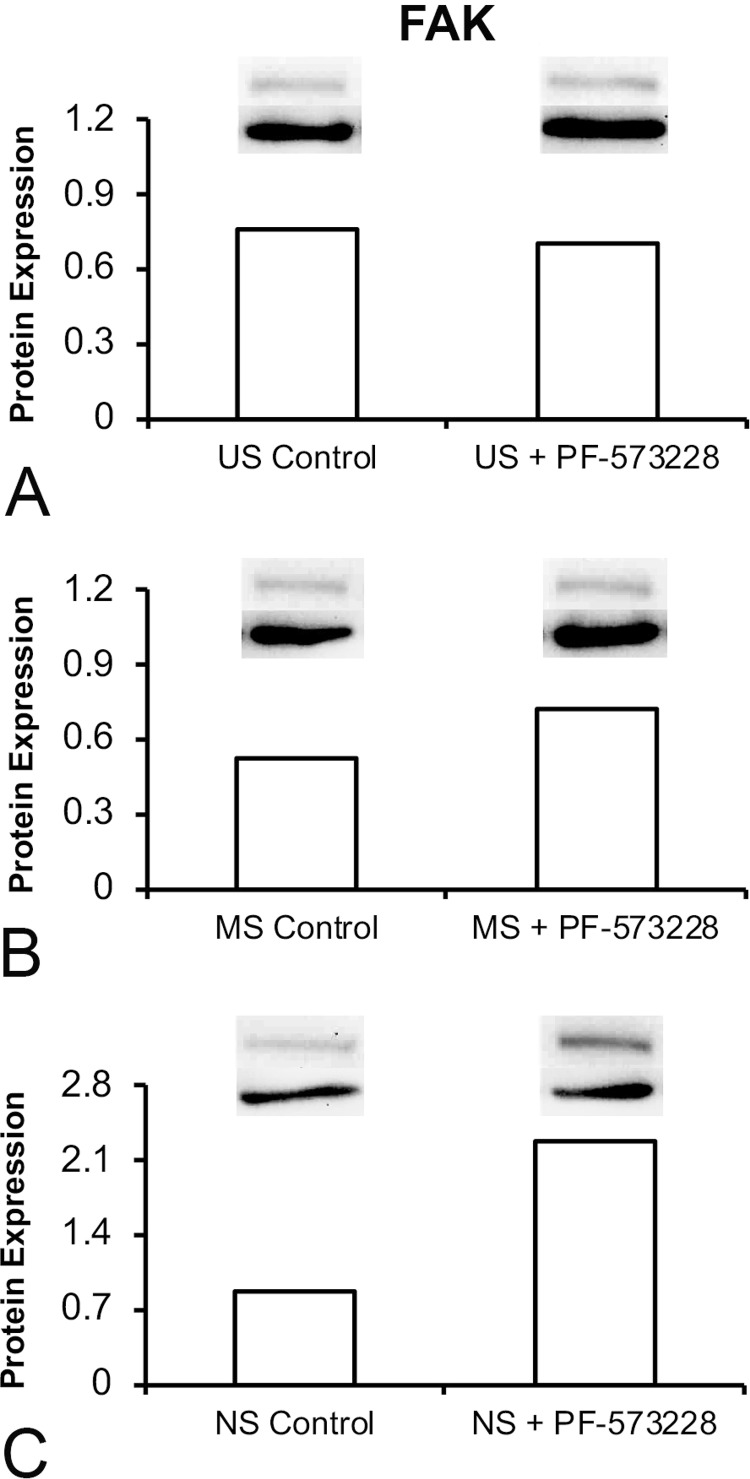
Protein expression of focal adhesion kinase (FAK) in cells cultured on US (A), MS (B), and NS (C) in the presence or absence of 0.1 µM PF-573228 on day 10

FAK inhibition slightly reduced FAK expression in cells grown on US (1.07-fold) ( Figure 8A ) but increased this expression in cells grown on MS and NS; of these two surfaces, a more pronounced effect was noted in the latter (1.3-fold and 2.5-fold, respectively) ( Figures 8B and 8C , respectively).

## Discussion

This study aimed to evaluate the participation of FAK in interactions between osteoblastic cells and Ti surface with three different topographies, namely, US, MS, and NS. The results indicated that FAK is relevant to osteoblastic differentiation of cells grown on Ti surfaces regardless of topographic characteristics since the inhibition of FAK reduced the differentiation of cells grown on all three surfaces. However, among the surfaces studied, NS was the surface in which the integrin signaling pathway was most affected by FAK inhibition.

Ti surface modification can improve implant wettability and increase the available surface for bone growth and fixation and blood clotting.^22^ Osseointegration of Ti depends mostly on the interactions between the material surface and cells, in which cell signaling pathways play an important role. FAK is involved in several signaling pathways due to its ability to bind to several proteins involved in these pathways; its function is related to many cellular processes such as migration, growth factor signaling, cell cycle progression, and cell survival.^26 , 27^ The present study focused on the role of an FAK inhibitor in the responses of osteoblastic cells to Ti. We selected an FAK inhibitor concentration of 0.1 μM because this value is the lowest concentration that did not significantly interfere with cell counting among the tested concentrations. This finding is supported by the observation that the same concentration of PF-573228 does not prevent cell proliferation but efficiently inhibits FAK activity.^20^

Cells grown on MS and NS showed a higher number of slender cytoplasmic projections attached to the surfaces compared with those grown on a smooth surface. In support of this finding, a previous study observed higher numbers of pseudopodia and more cell spreading on nanotextured Ti compared with those on a smooth surface.^28^ The FAK inhibitor reduced cell spreading on both US and MS surfaces without significantly affecting the morphology of cells grown on NS. This finding may be due to the higher wettability of NS compared with those of US and MS; such wettability could inactivate or compensate the inhibitory effect produced by the FAK inhibitor.^22 , 29^

In this study, FAK inhibition downregulated the gene expression of key bone markers and ALP activity in cells grown on all evaluated surfaces. FAK is a component of the focal adhesion complex and is essential for the development of integrin signaling. The participation of integrins in osteoblastic cell behavior has been extensively discussed in the literature. Indeed, we previously demonstrated the role of integrins α1, β1, and β3 in the osteogenic potential of NS.^10 , 11^ The use of an FAK inhibitor impaired the genotypic and phenotypic expression of osteoblasts cultured on all evaluated Ti surfaces, thereby corroborating a previous study showing that FAK inhibition adversely affects the development of osteoblastic phenotype in the same culture model used in the present work.^30^ Moreover, FAK inhibition increased the gene expression of integrins α1 and β1 in cells cultured on US and NS but not on MS. These data suggest the presence of a compensatory mechanism upregulating the expression of these integrins in response of FAK inhibition that is dependent on surface topography. In fact, this compensatory phenomenon has been previously described for other molecular mechanisms.^31 , 32^

The presence of PF-573228 did not affect the *Fak* gene expression of cells grown on US and MS but increased *Fak* gene and protein expression on NS. This finding may be explained by the positive modulation of the NS topography of FAK expression even in the presence of the FAK inhibitor and/or the ability of PF-573228 to physically bind FAK and inhibit its catalytic activity rather than its synthesis process. Previous studies using osteoblastic and fibroblastic cells revealed remarkable FAK expression and activation on nanostructured surfaces featuring 14 and 29 nm nanopits, similar to the pore size (22 nm on average) of the NS used in the present research^14 , 15 , 33 , 34^ .

Focal adhesion complexes are key structures participating in the interactions between cells and surfaces of biomaterials and may affect cell morphology, proliferation, differentiation, and apoptosis.^35^ Vinculin detection has been conducted to identify these complexes, but distinct data have been described^36 - 38^ . In the present work, we evaluated vinculin expression by immunofluorescence but we did not find a correspondence between the topography and vinculin expression of cells grown with or without the FAK inhibitor (data not shown). This result may be due to the effects of the trial periods chosen for the evaluation or the methodology used.^39^

## Conclusion

Our results demonstrated the relevance of FAK to the interactions between osteoblastic cells and Ti surfaces regardless of surface topography. We also observed that nanotopography upregulates FAK expression and integrin signaling pathway components during osteoblastic differentiation. Thus, the development of Ti surfaces with the ability to regulate FAK activity could positively impact the process of implant osseointegration.
